# Volumetric Food Quantification Using Computer Vision on a Depth-Sensing Smartphone: Preclinical Study

**DOI:** 10.2196/15294

**Published:** 2020-03-25

**Authors:** David Herzig, Christos T Nakas, Janine Stalder, Christophe Kosinski, Céline Laesser, Joachim Dehais, Raphael Jaeggi, Alexander Benedikt Leichtle, Fried-Michael Dahlweid, Christoph Stettler, Lia Bally

**Affiliations:** 1 Department of Diabetes, Endocrinology, Nutritional Medicine and Metabolism Bern University Hospital University of Bern Bern Switzerland; 2 Laboratory of Biometry University of Thessaly Nea Ionia/Volos, Magnesia Greece; 3 University Institute of Clinical Chemistry Inselspital, Bern University Hospital University of Bern Bern Switzerland; 4 Insel Data Science Center & Department for Technology and Innovation Bern University Hospital University of Bern Bern Switzerland

**Keywords:** depth camera, computer vision, dietary assessment, smartphone

## Abstract

**Background:**

Quantification of dietary intake is key to the prevention and management of numerous metabolic disorders. Conventional approaches are challenging, laborious, and lack accuracy. The recent advent of depth-sensing smartphones in conjunction with computer vision could facilitate reliable quantification of food intake.

**Objective:**

The objective of this study was to evaluate the accuracy of a novel smartphone app combining depth-sensing hardware with computer vision to quantify meal macronutrient content using volumetry.

**Methods:**

The app ran on a smartphone with a built-in depth sensor applying structured light (iPhone X). The app estimated weight, macronutrient (carbohydrate, protein, fat), and energy content of 48 randomly chosen meals (breakfasts, cooked meals, snacks) encompassing 128 food items. The reference weight was generated by weighing individual food items using a precision scale. The study endpoints were (1) error of estimated meal weight, (2) error of estimated meal macronutrient content and energy content, (3) segmentation performance, and (4) processing time.

**Results:**

In both absolute and relative terms, the mean (SD) absolute errors of the app’s estimates were 35.1 g (42.8 g; relative absolute error: 14.0% [12.2%]) for weight; 5.5 g (5.1 g; relative absolute error: 14.8% [10.9%]) for carbohydrate content; 1.3 g (1.7 g; relative absolute error: 12.3% [12.8%]) for fat content; 2.4 g (5.6 g; relative absolute error: 13.0% [13.8%]) for protein content; and 41.2 kcal (42.5 kcal; relative absolute error: 12.7% [10.8%]) for energy content. Although estimation accuracy was not affected by the viewing angle, the type of meal mattered, with slightly worse performance for cooked meals than for breakfasts and snacks. Segmentation adjustment was required for 7 of the 128 items. Mean (SD) processing time across all meals was 22.9 seconds (8.6 seconds).

**Conclusions:**

This study evaluated the accuracy of a novel smartphone app with an integrated depth-sensing camera and found highly accurate volume estimation across a broad range of food items. In addition, the system demonstrated high segmentation performance and low processing time, highlighting its usability.

## Introduction

Qualitative and quantitative assessment of dietary intake are cornerstones for the prevention and management of metabolic diseases such as obesity and diabetes [1,2]. Traditional manual food records that rely on human abilities to quantify food intake are time-consuming and error-prone [3]. One of the main challenges is the appropriate estimation of portion size (ie, volume) [4]. Inaccurate portion size estimation contributes up to 50% of the total estimation error [5]. Novel approaches replacing manual input by automated techniques may overcome the inherent limitations of traditional approaches, while increasing usability.

Mobile devices, currently ubiquitous, could simplify dietary monitoring. Although there are a number of commercially available apps offering access to food composition databases or providing reference images to facilitate estimation of portion size [6], they are generally limited by the need for manual user input.

High-quality smartphone cameras and computer vision approaches can be combined to fully automate portion size estimation. Users capture images of the meal using the smartphone camera, and the app subsequently builds a 3D model of the food to calculate its volume [7]. Combining the food volume with macronutrient-density databases, the app translates the volume into weight and then nutrient information. Food identification can be accomplished either by user selection or as part of the automated image processing, which further minimizes the need for user input [8].

Researchers have described several such systems [9-13]. A major challenge in many of these approaches lies in the capturing of the third dimension (depth) due to geometric constraints. In particular, factors such as precise food location, shape and size of food items, and changes in these parameters depending on camera perspective potentially interfere with reliable depth assessment. To overcome such constraints, fiducial markers, which ground the scene in a common frame of reference, are utilized. In addition, some systems use multiple images or video sequences of the food, followed by a complex calibration process. All these aspects inherently affect usability and accuracy.

The recent advent of miniaturized depth-sensing cameras embedded within smartphones (eg, iPhone X) opens a new horizon for automated food quantification. Using a single capture including depth information from any convenient viewing angle, this technology has the potential to eliminate the need for manual input, thereby increasing usability as well as accuracy. Therefore, the aim of this study was to evaluate the accuracy of a novel smartphone app that combines depth-sensing with computer vision to quantify food volume across a broad range of meals reflecting a real-life setting.

## Methods

### Study Design

The study occurred at the Central Kitchen Facility of the University Hospital Bern, Switzerland, in mid-January 2019. The system was tested on regular meals served to patients and hospital staff. A total of 48 test meals were randomly generated from a pre-defined pool of 128 food items. The test meals comprised the following meal types: breakfast, cooked meals, and snacks. Meals consisted of 4 (breakfast), 3 (cooked meals), or 1 (snacks) food items and were served on a standard plate or in a standard bowl. The meal content is described in Multimedia Appendix 1.

For each test meal, a single image was taken at a predefined angle of either 45° or 90° from the horizontal position (the angle was estimated by the user). The allocation of the capture angle of each meal was pre-defined using a balanced randomization procedure. The randomization sequence was produced as a binary sequence in three batches (by meal type). Images were captured under natural light conditions.

### Smartphone App

**Figure 1:**
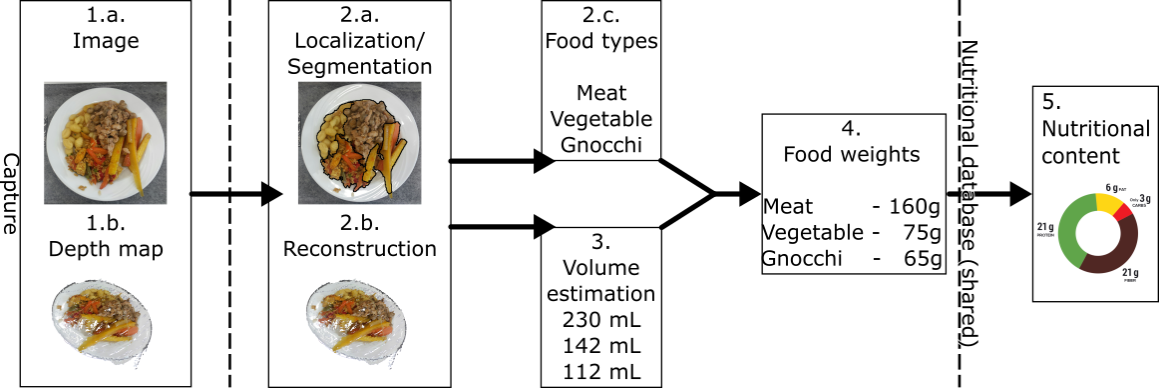
Automated food quantification workflow performed by the SNAQ app.

The app was provided by SNAQ GmbH (Zurich, Switzerland) using a software version from May 2018, and it was installed on an iPhone X (Apple Inc, Cupertino, California), which uses a built-in depth sensor applying structured light. The automated food quantification workflow is summarized in Figure 1 and consisted of (1) capturing the scene, (2) analyzing the scene, (3) estimating the volume, (4) converting the food volume into food weight, and (5) conversion of the food weight into macronutrient content.

First, the user takes a photograph using the phone, and a depth map of the food is generated through the phone’s front sensors consisting of a photo camera and an active depth sensor.

Second, the system partitions the image into consistent regions representing different items and eliminates those that are not food. To do so, a convolutional neural network has learned how food is structured in terms of sets of pixels and their correlations to the visual appearance of images. The data used to train the system consist of images with flags for each pixel indicating whether the respective pixel represents food. If the automated segmentation is not deemed satisfactory, the user can manually adjust the outlines of the items. A workflow of the segmentation as well as an example of good and bad segmentations are provided in Multimedia Appendix 2. Then, based on the depth map and input from the phone sensors, the visible point cloud is transformed into a set of surfaces using a Delaunay triangulation. The system extracts the location and orientation of the table (vertical plane) using the RANSAC-Algorithm [14] for an outlier-robust fitting. From this, the surfaces of each dish are defined. Selection of the food type is manually performed for each of the segmented food items.

Third, the segmented food items are used to cut the visible surface into partial food surfaces. Each food surface is then closed by the dish surfaces before their volume is calculated.

Fourth, the food volume is converted into food weight using a food density database. Finally, the food weight is converted into macronutrient content using the Swiss Food Composition Database [15].

This study was designed to assess the accuracy of automated quantification of portion sizes. Automated food recognition (ie, the taxonomy of the food items) was not a focus of this study. Instead, the user capturing the image selected the respective food item from a pre-defined list within the app.

### Reference Method

The reference weight was generated by weighing individual food items to the nearest 0.1 g using a precision scale (ME4002, Mettler Toledo, Greifensee, Switzerland). Conversion into macronutrient content was performed using the Swiss Food Composition Database [15].

### Endpoints

There were four study endpoints: (1) error of the estimated meal weight, (2) error of the estimated meal macronutrient content and energy content, (3) segmentation performance (defined as the number of items requiring manual correction of segmentation as well as intersection of the uncorrected and corrected segmentation areas over the corrected segmentation area), and (4) processing time (defined as the time period from image capture to macronutrient/energy output, including the time required for manual inputs).

### Sample Size Calculation and Statistical Analysis

The number of test meals in this study was determined based on a pilot experiment showing a mean (SD) difference in carbohydrate content of –2.6 g (9.2 g). Applying a power of 80% and significance level of .05 for 48 meals was deemed appropriate.

The error was determined on the meal level, and the following error metrics were used: bias, defined as the difference between estimation and reference (estimate-reference); absolute error, defined as |estimate-reference|; and 95% limits of agreement, calculated as ±2*SD of the bias. Bland-Altman plots were generated to visualize the level of agreement between the estimate and reference values. General linear models were used to assess the effect of meal type and inclination angle on the estimation error. *P* values <.05 were considered statistically significant. SPSS version 25.0 (IBM Corp, Armonk, NY) was used for statistical analysis. Data are described using mean (SD) and median (interquartile range [IQR]). All absolute error and bias values in this paper are presented as absolute values (g) followed by the relative values (%) in parentheses.

## Results

### Macronutrient and Energy Content of the Test Meals

The 48 test meals encompassed 128 food items. The mean reference macronutrient and energy contents of the 48 test meals are summarized in Multimedia Appendix 3*.* On average, the meals weighed 235.8 g (range 29.6-582.4 g). Meals contained an average 38.5 g carbohydrate (range 4.4-101.0 g), 14.6g protein (range 0.2-66.9 g), and 11.7 g fat (range 0.1-37.1 g). Mean energy content was 325 kcal (range 32-609 kcal). Insights into the study meals, including the representation of different meal types, are provided in Multimedia Appendix 4.

### Errors of Estimated Meal Weight, Estimated Meal Macronutrient Content, and Estimated Meal Energy Content

**Figure 2:**
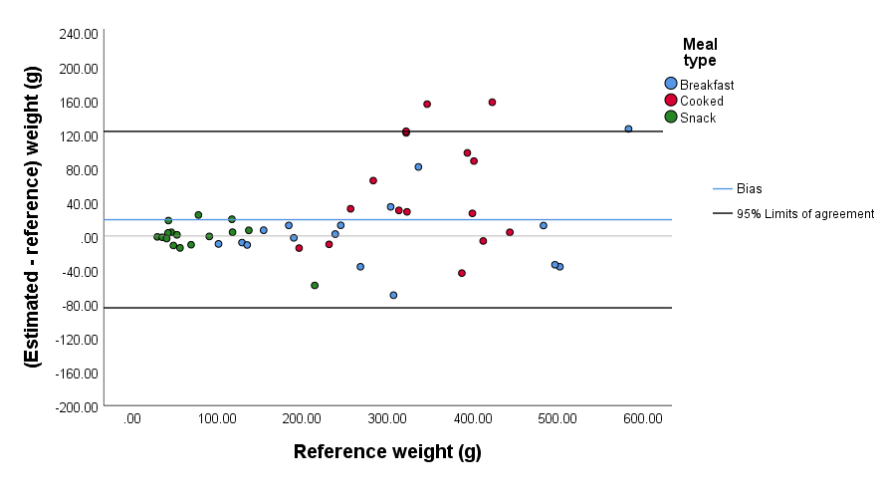
Bland-Altman plot illustrating the difference between the estimated and reference meal weights.

**Figure 3:**
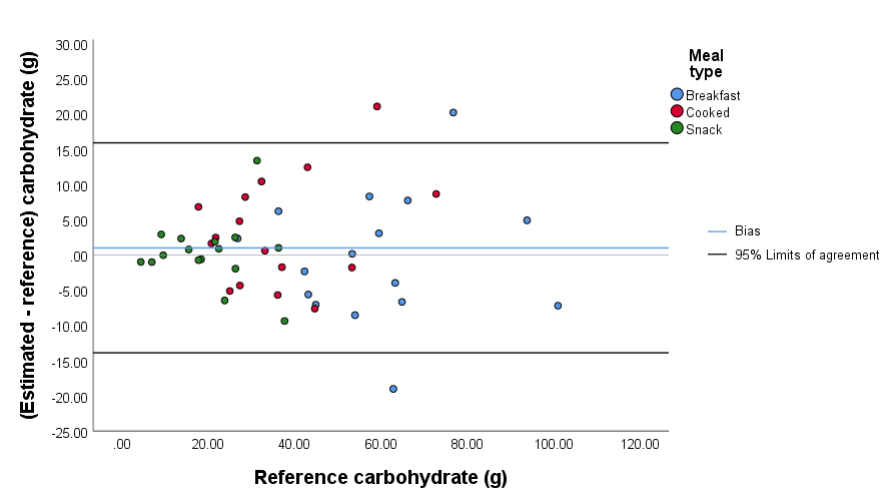
Bland-Altman plot illustrating the difference between the estimated and reference carbohydrate content of the meals.

**Figure 4:**
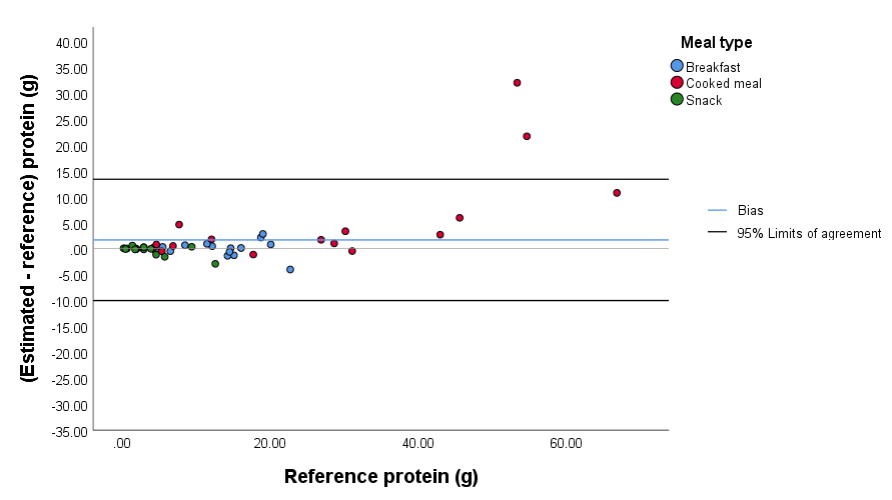
Bland-Altman plot illustrating the difference between the estimated and reference protein content of the meals.

**Figure 5:**
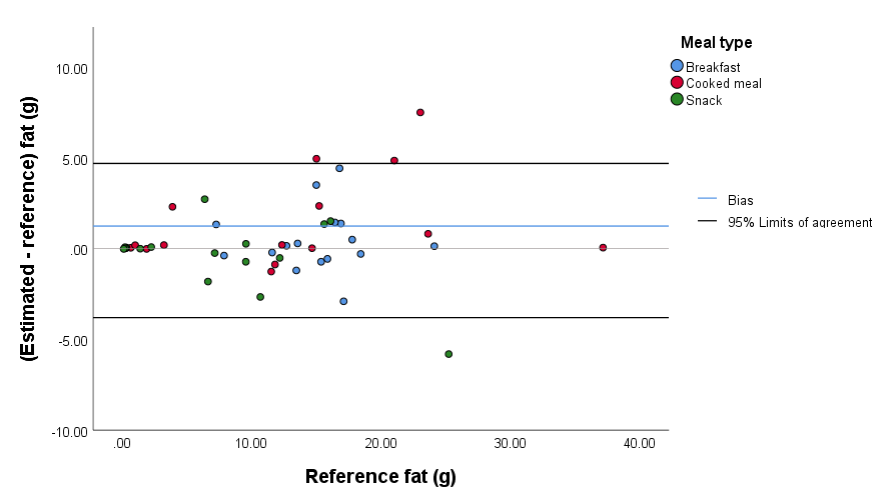
Bland-Altman plot illustrating the difference between the estimated and reference fat content of the meals.

**Figure 6:**
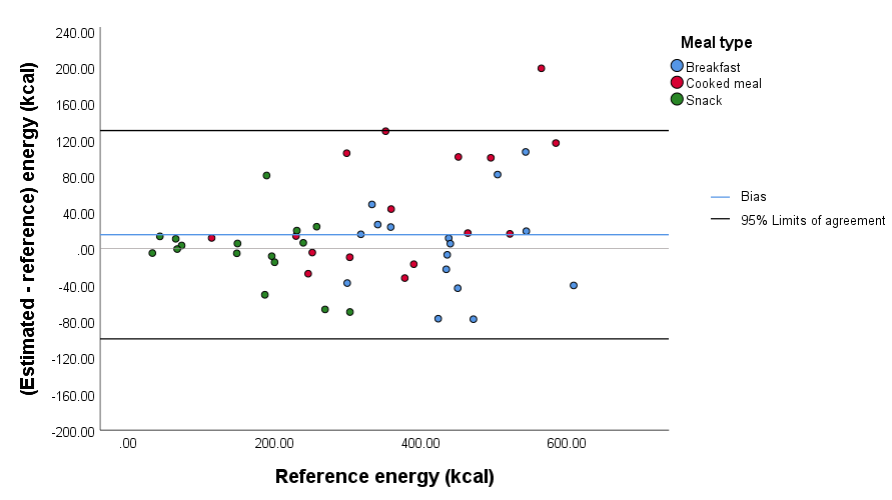
Bland-Altman plot illustrating the difference between the estimated and reference energy content of the meals.

The mean (SD) error metrics are summarized in Table 1, and the median (IQR) error metrics are presented in Multimedia Appendix 5. Corresponding Bland-Altman plots are presented in Figures 2-6. In both absolute and relative terms, the mean (SD) absolute error of the estimated weight for all meals was 35.1 g (42.8 g; 14.0% [12.2%]), and the mean (SD) bias was 19.3 g (52.1 g; 5.4% [17.8%]). The 95% limits of agreement were –84.8 g and 123.4 g (Figure 2).

**Table 1:** Error metrics for all meals and by meal, reported as the difference between the app’s estimate and the reference weight, macronutrient content, or energy content.

Meal characteristics		Absolute error				Bias				Limits of agreement, g	
		Absolute value, g, mean (SD)		Relative value, %, mean (SD)		Absolute value, g, mean (SD)		Relative value, %, mean (SD)			
**Weight**											
	All meals		35.1 (42.8)		14.0 (12.2)		19.3 (52.1)		5.4 (17.8)		–84.8, 123.4
	Breakfast		30.9 (34.6)		9.5 (7.5)		5.2 (46.8)		0.2 (12.3)		–88.3, 98.7
	Cooked meals		62.9 (53.8)		18.2 (14.7)		53.7 (63.6)		15.2 (18.0)		–73.4, 180.8
	Snacks		11.5 (14.6)		14.2 (12.4)		–1.0 (18.7)		1.0 (19.2)		–38.5, 36.4
**Carbohydrate**											
	All meals		5.5 (5.1)		14.8 (10.9)		1.0 (7.5)		2.9 (18.3)		–13.9, 15.9
	Breakfast		7.1 (5.5)		12.1 (7.9)		–0.5 (9.1)		–1.0 (14.7)		–18.7, 17.7
	Cooked meals		6.5 (5.2)		18.2 (11.6)		3.2 (7.8)		8.6 (20.3)		–12.4, 18.7
	Snacks		2.9 (3.7)		14.2 (12.5)		0.3 (4.8)		1.0 (19.2)		–9.3, 9.8
**Protein**											
	All meals		2.4 (5.6)		13.0 (13.8)		1.7 (5.9)		5.6 (15.2)		–10.0, 13.4
	Breakfast		1.0 (1.1)		7.3 (4.6)		0.0 (1.5)		0.0 (8.8)		–3.1, 3.1
	Cooked meals		5.6 (8.9)		17.4 (18.9)		5.3 (9.1)		15.3 (20.8)		–12.9, 23.5
	Snacks		0.5 (0.8)		14.2 (12.7)		–0.3 (0.9)		1.4 (19.4)		–2.0, 1.5
**Fat**											
	All meals		1.3 (1.7)		12.3 (12.8)		0.5 (2.1)		5.7 (16.9)		–3.8, 4.7
	Breakfast		1.2 (1.3)		8.4 (8.3)		0.4 (1.8)		3.1 (11.6)		–3.1, 3.9
	Cooked meals		1.6 (2.3)		14.4 (16.0)		1.3 (2.4)		12.1 (18.0)		–3.6, 6.2
	Snacks		1.1 (1.6)		14.0 (12.8)		–0.4 (1.9)		1.8 (19.2)		–4.1, 3.5
**Energy**											
	All meals		41.2 (42.5)^a^		12.7 (10.8)		15.5 (57.4)^a^		4.1 (16.2)		–99.4, 130.3^a^
	Breakfast		40.4 (30.5)^a^		9.2 (6.2)		2.1 (51.6)^a^		0.4 (11.4)		–101.1, 105.4^a^
	Cooked meals		59.1 (58.0)^a^		14.7 (12.3)		47.8 (68.2)^a^		11.0 (15.8)		–88.7, 184.2^a^
	Snacks		24.1 (26.9)^a^		14.2 (12.4)		–3.5 (36.5)^a^		1.0 (19.2)		–76.4, 69.5^a^

^a^kcal.

The mean (SD) absolute error of the estimated carbohydrate content for all meals was 5.5 g (5.1 g; 14.8% [10.9%]), and the mean (SD) bias was 1.0 g (7.5 g; 2.9% [18.3%]). The 95% limits of agreement were –13.9 g and 15.9 g (Figure 3). The mean (SD) absolute error of the estimated protein content for all meals was 2.4 g (5.6 g; 13.0% [13.8%]), and the mean (SD) bias was 1.7 g (5.9 g; 5.6% [18.2%]). The 95% limits of agreement were –10.0 g and 13.4 g (Figure 4). The mean (SD) absolute error of the estimated fat content for all meals was 1.3 g (1.7 g; 12.3% [12.8%]), and the mean (SD) bias was 0.5 g (2.1 g; 5.7% [16.9%]). The 95% limits of agreement were –3.8 g and 4.7 g (Figure 5). The mean (SD) absolute error of the estimated energy content for all meals was 41.2 kcal (42.5 kcal; 12.7% [10.8%]), and the mean (SD) bias was 15.5 kcal (57.4 kcal; 4.1% [16.2%]). The 95% limits of agreement were –99.4 kcal and 130.3 kcal (Figure 6).

While viewing angle had no significant influence on the accuracy of macronutrient and energy estimation (*P*=.96, *P*=.83, *P*=.99, *P*=.73, and *P*=.70 for absolute errors in weight, carbohydrate, protein, fat, and energy content, respectively), we observed a significant effect of meal type on the accuracy for all macronutrients and energy content (*P*=.001, *P*=.001, *P*=.001, *P*=.002, and *P*=.005 for absolute errors in weight, carbohydrate, protein, fat, and energy content, respectively). The mean bias values of the cooked meals for carbohydrate, protein, and fat content were significantly higher than for the breakfasts, with a mean (SD) difference in bias of 18.3% (4.9%) for carbohydrate, 19.6% (4.9%) for protein, and 17.7% (5.1%) for fat (all *P*<.001). The comparison of bias for snacks relative to the bias of cooked meals resulted in marginal outcomes for fat (*P*=.02), carbohydrate (*P*=.08), and protein (*P*=.07). Furthermore, the bias for snacks was not different from the bias for breakfasts for all macronutrients (*P*=.37, *P*=.26, *P*=.23, *P*=.79, and *P*=.50 for weight, carbohydrate, protein, fat, and energy content, respectively).

### Segmentation Performance

In 7 of the 128 items (5.5%), segmentation required manual adjustment. The intersection over union of unadjusted to adjusted segmentation area was 71.8%.

### Processing Time

**Figure 7:**
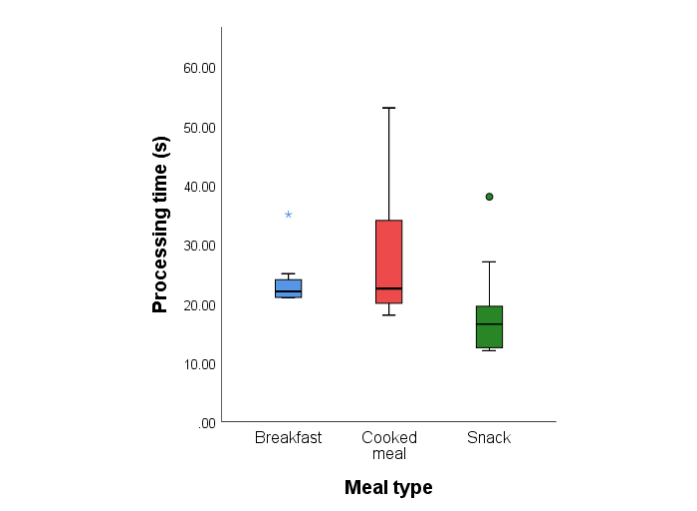
Box-plot of the processing time according to meal type. Box-plots show median values (solid line), interquartile range (IQR; box outline), spread of data points without outliers (whiskers) and outliers identified as 1.5*IQR (symbols).

Mean (SD) processing time across all meal types was 22.9 seconds (8.6 seconds). Processing time was significantly lower for snacks (mean 17.9 seconds, SD 7.0 seconds) compared with cooked meals (mean 27.8 seconds, SD 10.8 seconds; mean difference –9.9 seconds, SD 2.7 seconds; *P*<.001). Processing time was lower for breakfast (mean 23.1 seconds, SD 3.5 seconds) compared with cooked meals (mean difference –4.7 seconds, SD 2.8 seconds; *P*=.12). Figure 7 provides the processing time stratified by meal type.

## Discussion

This study evaluated the accuracy of a novel smartphone app that combines depth sensing with computer vision using volumetry to quantify the macronutrient content of meals in a real-life setting. The main findings were as follows: the accuracy was adequate across all macronutrients, the accuracy differed according to meal type (lower for cooked meals than for snacks and breakfast), segmentation was good, and processing was fast.

When compared with previous reports of apps using computer vision without depth sensors, the present app had comparable, or even superior, accuracy. Rhyner et al [16] reported a mean absolute error of 26.2% in carbohydrate content when assessing 60 cooked meals with non-overlapping food items. In a further preclinical study assessing the accuracy of the prototype used by Rhyner et al and based on 54 cooked meals, the mean absolute error in carbohydrate quantification was 14.8 g, which corresponds to 24.7% for a meal carbohydrate content of 60 g [17]. In contract, with the app in the present study, mean absolute errors in macronutrient content estimation ranged from 12.3% (fat) to 15% (carbohydrate).

Of note, two recent studies assessing the accuracy of image–based food quantification using volume as a reference metric reported mean absolute errors in volume estimation of 7.2% [12] and 5.8% [18] based on the assessment of 5 and 20 food items, respectively. These slightly smaller errors compared to those in this study can be explained by the different reference metric used to define the system accuracy (error in estimated volume versus error in estimated weight and consequently macronutrient content). Of note, errors in weight estimation have two potential sources: inaccuracies in volume and density estimation. Additionally, operational aspects of the previously reported systems differ from those in this study. Xu et al [12] used a complex multi-step approach including reference objects, while Makhsous et al [13] added a depth sensor with structured light to the smartphone and complemented their approach with video sequences, significantly increasing the complexity of the workup. These differences highlight the important tradeoffs between accuracy and usability.

Of note, this study revealed a comparably short processing time, ranging from 18 seconds for snacks to 29 seconds for cooked meals. This is faster than those reported in previous studies, where processing times generally exceeded the limit of 1 minute [19]. This highlights the usability of the present system even when applied to meals in a real-life setting.

The accuracy of the tested app differed according to meal type and was lower for cooked meals than for breakfasts and snacks. This might have resulted from the different levels of complexity in terms of scene analysis of the respective meals. Whereas the breakfasts and snacks had food items that were clearly separated from each other, the cooked meals had food items with touching borders or a certain degree of overlap. Notably, the angle of image capture did not affect the estimation accuracy in this study, indicating the flexibility, usability, and robustness of the system.

We acknowledge a number of limitations of this study. First, the assessment was limited to meals provided by the hospital kitchen, preventing a generalized statement on the accuracy. However, the system was tested using real-life meals, underscoring its potential use in practice. Second, the system was limited to a single type of smartphone (iPhone X) with a depth sensor, precluding statements on the performance of the software combined with different hardware components. However, this approach supports the strength of providing a commercially available tool. Third, the depth sensor limited the reconstruction to 1/20^th^ of the resolution and with lower depth precision than with a passive depth sensor (dual camera approach). However, the use of a depth sensor foregoes the need for fiducial markers, rendering it more convenient to users. Fourth, we served all meals on one plate or bowl type, possibly reducing the variation in volume estimation that was unrelated to the depth sensor. Finally, this study exclusively focused on the accuracy of volume quantification and did not consider food recognition.

When considering both observed accuracy and usability of the present system, the field of potential use appears broad. Such a system may be of interest in the medical sector to assist with nutritional counseling and management of patients with metabolic disorders (eg, diabetes mellitus, obesity) or at risk of malnutrition. Beyond this, such a system may be valuable in nutritional epidemiology due to the potential to systematically and accurately monitor dietary intake on a large scale.

In conclusion, this study evaluated the accuracy of a novel smartphone app with integrated depth sensing and found a high level of accuracy in volumetric macronutrient and energy estimation across a broad set of meals in a real-life setting. In addition, the system demonstrated high segmentation performance and low processing time, highlighting its usability.

## Appendix

Multimedia Appendix 1 Food items assessed using the SNAQ smartphone app.

Multimedia Appendix 2 Examples of good and bad segmentation by the SNAQ app during test meal analysis.

Multimedia Appendix 3 Mean macronutrient and energy content of the tested meals.

Multimedia Appendix 4 Examples of the assessed meal types.

Multimedia Appendix 5 Estimation accuracy, as determined using the error metrics of the analyzed meals.

## Abbreviations

IQR: interquartile range.
