# Supplementary material for: Volumetric Food Quantification Using Computer Vision on a Depth-Sensing Smartphone: Preclinical Study
Source: JMIR Mhealth Uhealth. 2020 Mar 25;8(3):e15294. doi: 10.2196/15294 (PMC7142738; doi:10.2196/15294)
Supplement: Multimedia Appendix 1 [file mhealth_v8i3e15294_app1.docx]

| **Supplementary Table 1. Food items** | |  |
| --- | --- | --- |
| **Breakfast** | **Cooked Meals** | **Snacks** |
| Banana | Beef balls | Apple |
| Birchwermüesli | Bratwurst | Apple slices |
| Blueberries | Breaded cutlet | Applesauce |
| Bread (bun) | Carrots | Balisto |
| Bread (multigrain) | Chicken breast | Bricelet |
| Bread (whole-grain without crust) | Cucumbers salad | Chocolate bar |
| Bread (whole-grain) | Curly endive | Darvida |
| Butter | Eisberg lettuce | Grapes |
| Cheese (Babybel) | Fried potatoes | Kägi Fret |
| Cheese (Camembert) | Hummus | Mandarin |
| Cheese (Emmental) | Mashed potatoes | Mars chocolate bar |
| Cheese (Gruyère) | Pepperoni | Milky-Way |
| Cheese spread (1/4 fat) | Red cabbage | Ragusa |
| Cheese spread (full fat) | Rice | Salted peanuts |
| Cornflakes | Roast veal | Twix |
| Croissants | Salt potatoes | Quark Light (Apricot) |
| Fruit salad | Sliced turkey |  |
| Honey | Spaghetti |  |
| Jam (Strawberry) | Spinach |  |
| Jelly (Apple) | Zuchini |  |
| Jelly (Quinces) |  |  |
| Margarine |  |  |
| Nutella |  |  |
| Orange |  |  |
| Quark (Strawberry) |  |  |
| Rice pudding |  |  |
| Rusk (Zwieback) |  |  |
| Semolina |  |  |
| Vanilla porridge |  |  |
| Watermelon |  |  |
| Yoghurt (ananas) |  |  |
| Yoghurt (Mocca) |  |  |
| Yoghurt (Nature) |  |  |
| Yoghurt (strawberry) |  |  |
|  |  |  |
