# Supplementary figures and images for: Volumetric Food Quantification Using Computer Vision on a Depth-Sensing Smartphone: Preclinical Study
Source: JMIR Mhealth Uhealth. 2020 Mar 25;8(3):e15294. doi: 10.2196/15294 (PMC7142738; doi:10.2196/15294)

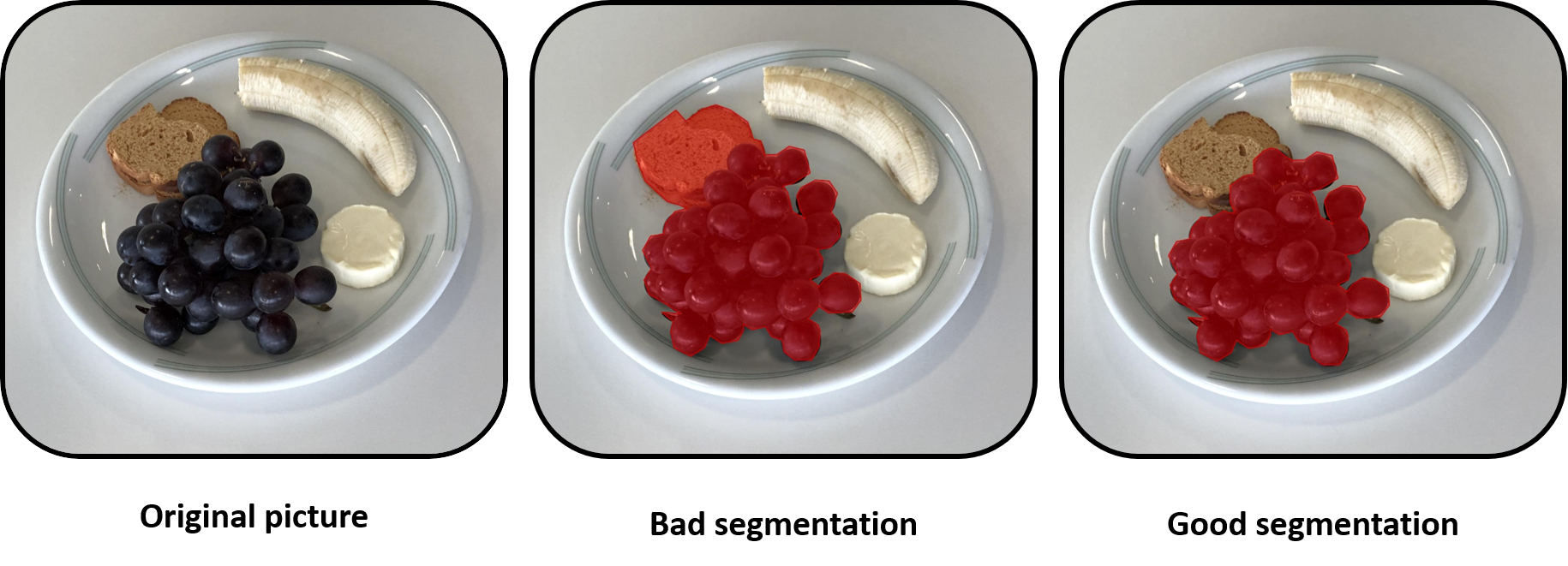

Supplement: Multimedia Appendix 2 [file mhealth_v8i3e15294_app2.png]

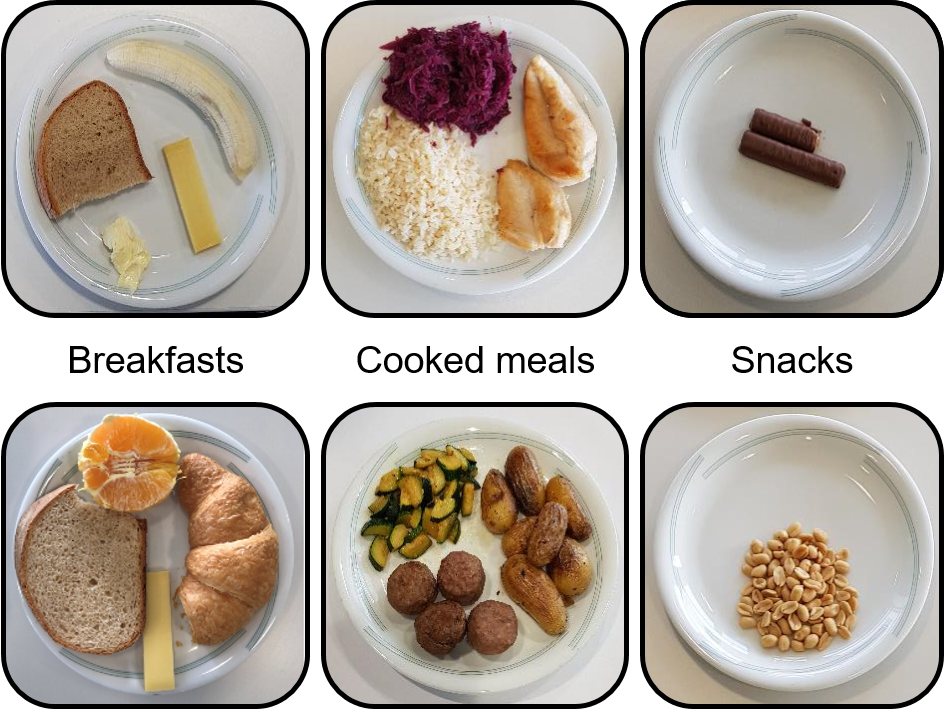

Supplement: Multimedia Appendix 4 [file mhealth_v8i3e15294_app4.png]
