# Supplementary material for: Volumetric Food Quantification Using Computer Vision on a Depth-Sensing Smartphone: Preclinical Study
Source: JMIR Mhealth Uhealth. 2020 Mar 25;8(3):e15294. doi: 10.2196/15294 (PMC7142738; doi:10.2196/15294)
Supplement: Multimedia Appendix 3 [file mhealth_v8i3e15294_app3.docx]

|  |  | Weight (g) | Carbohydrate (g) | Protein (g) | Fat (g) | Energy (kcal) |
| --- | --- | --- | --- | --- | --- | --- |
| Total (n=48) | Mean | 235.8 | 38.5 | 14.6 | 11.7 | 330.0 |
|  | SD | 152.4 | 22.1 | 15.7 | 8.1 | 168.1 |
|  | Range | (29.6-582.4) | (4.4-101.0) | (0.2-66.9) | (0.1-37.1) | (32.5-840.7) |
| Breakfast (n=16) | Mean | 290.8 | 59.1 | 13.0 | 15.0 | 433.7 |
|  | SD | 151.2 | 19.6 | 5.8 | 4.1 | 88.5 |
|  | Range | (101.4-582.4) | (26.8-101.0) | (4.5-22.7) | (7.3-24.4) | (299.0-608.5) |
| Cooked meals (n=16) | Mean | 340.5 | 36.2 | 27.4 | 12.3 | 374.8 |
|  | SD | 72.8 | 15.1 | 20.4 | 10.4 | 132.4 |
|  | Range | (195.9-443.1) | (17.7-72.8) | (4.3-66.9) | (0.3-37.1) | (113.5-585.2) |
| Snacks (n=16) | Mean | 76.1 | 20.0 | 3.4 | 7.7 | 165.2 |
|  | SD | 49.3 | 10.0 | 3.4 | 7.2 | 85.6 |
|  | Range | (29.6-214.2) | (4.4-37.7) | (0.2-12.6) | (0.1-25.2) | (32.5-302.6) |
