# Supplementary material for: Volumetric Food Quantification Using Computer Vision on a Depth-Sensing Smartphone: Preclinical Study
Source: JMIR Mhealth Uhealth. 2020 Mar 25;8(3):e15294. doi: 10.2196/15294 (PMC7142738; doi:10.2196/15294)
Supplement: Multimedia Appendix 5 [file mhealth_v8i3e15294_app5.docx]

| **Supplementary Table 3: Estimation accuracy** | |  |
| --- | --- | --- |
|  | Absolute error (Median [IQR]) | Bias (Median [IQR]) |
| ***Weight*** |  |  |
| Total | 14.1g [6.5-38.1g ] (9.0% [4.8-22.8%]) | 4.4g [-9.4-29.0] (3.3% [-6.9-13.6%]) |
| Breakfast | 12.6g [8.9-36.2g] (7.0% [5.0-11.9%]) | 0.1g [-16.4-12.6] (-0.1% [-7.4-5.6%]) |
| Cooked Meals | 38.0g [23.6-104.0g] (11.9% [7.1-28.1%]) | 31.2g [1.8-104.0] (11.1% [0.4-28.1%]) |
| Snacks | 5.6g [2.6-15.1g] (9.1% [4.1,23.5%]) | 0.5g [-4.8-5.1] (1.1% [-9.3-8.9%]) |
| ***Carbohydrate*** |  |  |
| Total | 4.6g [1.8-7.7g] (12.5% [5.5-21.2%]) | 0.7g [-4.1-4.8] (2.3% [-11.0-12.5%]) |
| Breakfast | 6.4g [3.8-7.9g] (11.0% [6.1-15.8%]) | -1.1g [-6.8-5.2] (-2.6% [-11.0-9.4%]) |
| Cooked Meals | 5.4g [2.3-8.3g] (16.5% [10.5-28.8%]) | 2.0g [-2.4-8.3] (9.6% [-7.5-28.8%]) |
| Snacks | 1.5g [0.8-2.6g] (9.1% [4.1-23.6%]) | 0.4g [-1.0-2.0] (1.1% [-9.3-8.9%]) |
| ***Protein*** |  |  |
| Total | 0.6g [0.2-1.7g] (8.1% [4.8-15.3%]) | 0.2g [-0.3-1.0] (0.0% [-0.1-1.1%]) |
| Breakfast | 0.6g [0.3-1.3g] (6.8% [4.2-8.7%]) | 0.1g [-0.5-0.7] (0.0% [-0.1,0.1%]) |
| Cooked Meals | 1.8g [0.7-5.0g] (10.3% [6.4-16.4%]) | 1.8g [0.5-5.0] (0.1% [0.0-0.2%]) |
| Snacks | 0.1g [0.1-0.4g] (9.2% [4.3-23.5%]) | 0.0g [-0.1-0.1] (0.0% [-0.1-0.1%]) |
| ***Fat*** |  |  |
| Total | 0.5g [0.2-1.6g] (7.5% [3.2-18.5%]) | 0.1g [-0.3-1.3] (2.5% [-3.7-11.9%]) |
| Breakfast | 0.6g [0.3-1.4g] (4.8% [2.1-10.9%]) | 0.2g [-0.4-1.4] (0.9% [-3.8-8.4%]) |
| Cooked Meals | 0.5g [0.1-2.3g] (9.0% [3.0-19.8%]) | 0.2g [0.0-2.3] (6.8% [0.2-19.8%]) |
| Snacks | 0.4g [0.1-1.6] (8.1% [4.1-23.5%]) | 0.0g [-0.6-0.1] (1.4% [-7.2-8.9%]) |
| ***Energy*** |  |  |
| Total | 23.3kcal [11.5-67.6kcal] (9.1% [4.1-18.6%]) | 8.7kcal [-15.5-24.9kcal] (3.3% [-5.6-12.8%]) |
| Breakfast | 32.2kcal [18.4-55.9kcal] (7.2% [4.6-15.1%]) | 8.6kcal [-38.5-24.5kcal] (2.0% [-7.4-6.9%]) |
| Cooked Meals | 30.0kcal [15.8-102.2kcal] (10.8% [4.2-20.8%]) | 16.8kcal [-5.5-102.2kcal] (8.3% [-2.0-20.8%]) |
| Snacks | 12.3kcal [5.6-30.9kcal] (9.1% [4.1-23.5%]) | 1.6kcal [-9.9-11.6kcal] (1.1% [-9.3-8.9%]) |
| Error metrics related to the difference between the application’s estimate and the reference weight, macronutrient (carbohydrate, protein, fat) in grams (g). Errors are presented across all meal types (total) and stratified according to meal type. IQR, Interquartile range | | |
